# Mining characteristics of epidemiological studies from Medline: a case study in obesity

**DOI:** 10.1186/2041-1480-5-22

**Published:** 2014-05-19

**Authors:** George Karystianis, Iain Buchan, Goran Nenadic

**Affiliations:** 1 School of Computer Science, University of Manchester, Kilburn Building, Oxford Road, Manchester, UK; 2 Health e-Research Centre, Manchester, UK; 3 Centre for Health Informatics, Institute of Population Health, University of Manchester, Manchester, UK

**Keywords:** Text mining, Epidemiology, Key characteristics, Rule-based methodology

## Abstract

**Background:**

The health sciences literature incorporates a relatively large subset of epidemiological studies that focus on population-level findings, including various determinants, outcomes and correlations. Extracting structured information about those characteristics would be useful for more complete understanding of diseases and for meta-analyses and systematic reviews.

**Results:**

We present an information extraction approach that enables users to identify key characteristics of epidemiological studies from MEDLINE abstracts. It extracts six types of epidemiological characteristic: design of the study, population that has been studied, exposure, outcome, covariates and effect size. We have developed a generic rule-based approach that has been designed according to semantic patterns observed in text, and tested it in the domain of obesity. Identified exposure, outcome and covariate concepts are clustered into health-related groups of interest. On a manually annotated test corpus of 60 epidemiological abstracts, the system achieved precision, recall and F-score between 79-100%, 80-100% and 82-96% respectively. We report the results of applying the method to a large scale epidemiological corpus related to obesity.

**Conclusions:**

The experiments suggest that the proposed approach could identify key epidemiological characteristics associated with a complex clinical problem from related abstracts. When integrated over the literature, the extracted data can be used to provide a more complete picture of epidemiological efforts, and thus support understanding via meta-analysis and systematic reviews.

## Background

Epidemiological studies aim to discover the patterns and determinants of diseases, and other health related states by studying the health of populations in standardised ways. They are valuable sources of evidence for public health measures and for shaping of research questions in the clinical and biological aspects of complex diseases. Nevertheless, the increasing amount of published literature leads to information overload, making the task of reading and integrating relevant knowledge a challenging process [1-3]. For example, there are more than 23,000 obesity-related articles reporting on different epidemiological findings, including almost 3,000 articles with *obesity/epidemiology* as a MeSH descriptor in 2012, with more than 15,000 such articles in the last 10 years. Therefore, there is a need for systems that enable the extraction of salient epidemiological study features in order to assist investigators to reduce the time required to detect, summarise and incorporate epidemiological information from the relevant literature [4].

Epidemiology is a relatively structured field with its own dictionary and reporting style, deliberately written in a typical semi-structured format in order to standardize and improve study design, communication and collaboration. The standard characteristics in most epidemiological studies include [5]:

• *study design* - a specific plan or protocol that has been followed in the conduct of the study;

• *population* - demographic details of the individuals (e.g., gender, age, ethnicity, nationality) participating in an epidemiological study;

• *exposure* - a factor, event, characteristic or other definable entity that brings about change in a health condition or in other defined characteristics;

• *outcome* - the consequence from the exposure in the population of interest;

• *covariate* - a concept that is possibly predictive of the outcome under study;

• *effect size* - the measure of the strength of the relationship between variables, that relates outcomes to exposures in the population of interest.

In this paper we present a system that enables the identification and retrieval of the key characteristics from the epidemiological studies. We have applied the system to the obesity epidemiological literature. Obesity is one of the most important health problems of the 21^st^ century [6], presenting a great public health and economic challenge [7-9]. The rapid and worldwide spread of obesity has affected people of all ages, genders, geographies and ethnicities. It has been regarded as a multi-dimensional disorder [10], with major behavioural and environmental determinants, with genetics playing only a minor role [7].

### Related work

In the last decade, a significant amount of research has been performed on the extraction of information in the biomedical field, especially on the identification of biological [11,12] and clinical concepts [13,14] in the literature. In clinical text mining, several attempts have been made to extract various kinds of information from case studies and clinical trials in particular [1-4,15-23]. For example, De Bruijn et al. [22] applied text classification with a “weak” regular expression matcher on randomized clinical trial (RCT) reports for the recognition of key trial information that included 23 characteristics (e.g. eligibility criteria, sample size, route of treatment, etc.) with overall precision of 75%. The system was further expanded to identify and extract specific characteristics such as primary outcome names and names of experimental treatment from journal articles reporting RCTs [4], with precision of 93%. However, they focused solely on RCTs and especially on randomized controlled drug treatment trials. Hara and Matsumoto [1] extracted information about the design of phase III clinical trials. They extracted patient population and compared associated treatments through noun phrase chunking and categorisation along with regular expression pattern matching. They reported precision for population and compared treatments of 80% and 82% respectively. Hansen et al. [2] worked on RCTs identifying the numbers of the trial participants through a support vector machine algorithm with 97% precision, while Fizman et al. [19] aimed to recognize metabolic syndrome risk factors in MEDLINE citations through automatic semantic interpretation with 67% precision. However, to the best of our knowledge, there is no approach available for recognising key information elements from various types of epidemiological studies that are related to a particular health problem.

## Methods

**Figure 1:**
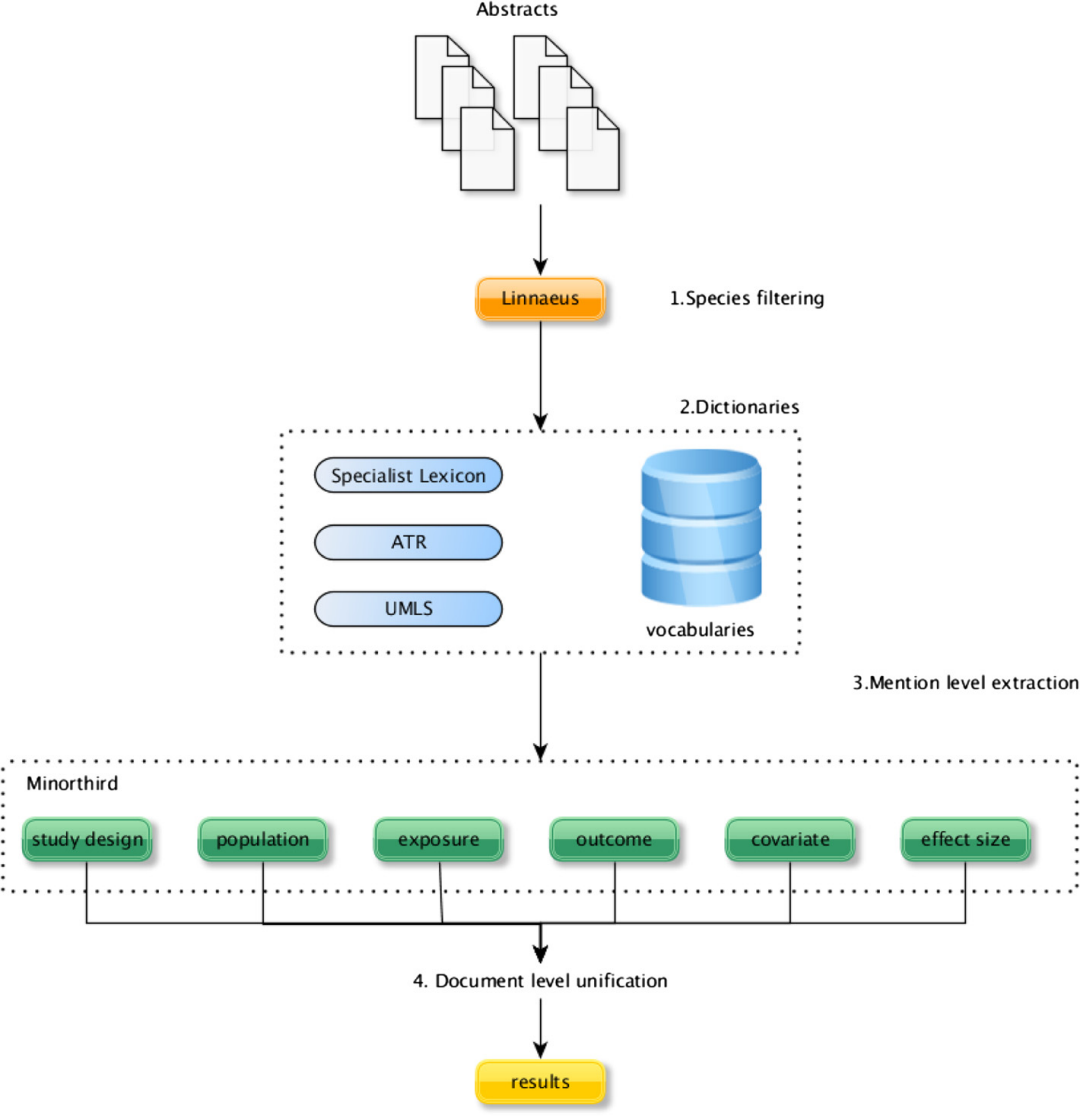
**The four steps of the approach applied to epidemiological abstracts in order to recognise key characteristics.** Linnaeus is used to filter out abstracts not related to humans; Dictionary look-up and automatic term recognition (ATR) are applied to identify major medical concepts in text; MinorThird is used as an environment for the rule application and mention identification of epidemiological characteristics.

Our approach involved the design and implementation of generic rule-based patterns, which identify mentions of particular characteristics of epidemiological studies in PubMed abstracts (Figure 1). The rules are based on patterns that were engineered from a sample of 60 epidemiological abstracts in the domain of obesity. Mentions of six semantic types (study design, population, exposures, outcomes, covariates and effect size) have been manually identified and reviewed. Additionally, a development set with additional 30 abstracts was used to optimise the performance of the rules. These steps are explained here in more details.

1. **Abstract selection and species filtering.** In the first step, abstracts are retrieved from PubMed using specific MeSH terms (e.g. *obesity/epidemiology[mesh]*). They are checked by LINNAEUS, a species identification system [24], to filter out studies based on non-human species.

2. **Building of dictionaries of potential mentions.** In the second step, a number of semantic classes are identified using custom-made vocabularies that include terms to detect key characteristics in epidemiological study abstracts (e.g. dictionaries of words that indicate tudy design, population totals, etc. – a total of fourteen dictionaries). We also identify mentions of Unified Medical Language System (UMLS) [25] terms and additionally apply the Specialist lexicon[26] in order to extract potential exposure, outcome, covariate and population concepts. Finally, epidemiological abstracts are processed with an automatic term recognition (ATR) method for the extraction of multi-word candidate concepts and their variants [27,28]. Filtering against a common stop-word list (created by Fox [29]) is applied to remove any concepts of non-biomedical nature.

3. **Mention-level application of rules.** In the third step, rules are applied to the abstracts for each of the six epidemiological characteristic separately. The rules make use of two constituent types: frozen lexical expressions (used as anchors for specific categories) and specific semantic classes identified through the vocabularies (identified in step 2), which are combined using regular expressions. The frozen lexical expressions can contain particular verbs, prepositions or certain nouns. Table 1 shows the number of rules created for each of the six characteristics with some typical examples. As a result of the application of rules, *candidate mentions* of epidemiological concepts are tagged in text. We used MinorThird [30] for annotating and recognizing entities of interest.

4. **Document-level unification.** Finally, in cases where several candidate mentions for a single epidemiological characteristic were recognised in a given document, we also ‘unified’ them to get document-level annotations using the following approach: if a given mention is part of a longer mention, then we select only the longer. Mentions that are not included in other mentions (of the same type) are also returned. In addition, where applicable (i.e. for exposures, outcomes and covariates), these mentions are mapped to one of the 15 UMLS semantic groups (Activities and Behaviors, Anatomy, Chemicals and Drugs, Concepts and Ideas, Devices, Disorders, Genes and Molecular, Geographic Areas, Living Beings, Objects, Occupations, Organizations, Phenomena, Physiology and Procedures). We decided to perform the mapping to high-level UMLS semantic groups to assist epidemiologists in the application of an ‘epidemiological sieve’, which could help them decide whether or not to include abstracts for more detailed inspection. For example, highlighting different types of determinant (e.g. *demographic* vs. *lifestyle*) would be useful for considering the completeness and relevance of factors in a particular study by emphasizing possible connections between the background of the exposure and/or the outcomes.

**Table 1:** Examples of rules for recognition of study design, population, exposure, outcome, covariate and effect size in epidemiological abstracts

**Characteristic (number of rules)**	**Examples**	**Identified span (in bold)**					
**Study design** (16 rules)	**Rule**	[@st			a(types)]		
	Methods: This was a **cross-sectional study** of 214 overweight/obese …	**cross-sectional**			**study**		
**Population** (119 rules)	**Rule**	a(totals)	re(‘(of|on|in)’)		[@stats		a(clusters)]
	Sibling study in a prospective cohort of **208,866 men** from …	cohort	of		**208,866**		**men**
	**Rule**	@multiple	re(‘with|in|on’)?		[a(clusters)	re(‘with|without’)	@multiple]
	bone mineral density in **patients with type 2 diabetes**	bone mineral density	in		**patients**	**with**	**type 2 diabetes**
**Exposure** (134 rules)	**Rule**	a(relations)	eq(‘between’)		[@multiple]	eq(‘and’)	@multiple
	… and analyze the association between **body mass index** and blood pressure in …	association	Between		**body mass index**	and	blood pressure
	**Rule**	[@multiple]	a(be)	a(related)	a(with)	eq(‘onset’)?	eq(‘of’)?
	**Short sleep duration** is associated with onset of obesity	**Short sleep duration**	is	associated	with	onset	of
**Outcome** (100 rules)	**Rule**	@factors	eq(‘of’)			[@multiple]	
	Cardiovascular and disease related predictors of **depression**	predictors	of			**depression**	
	**Rule**	@multiple	a(be)	a(adverbs)	a(related)	a(with)	[@multiple]
	Conclusions coffee intake is inversely associated with **t2dm** in Chinese.	coffee intake	is	inversely	associated	with	**t2dm**
**Covariate** (28 rules)	**Rule**	a(adj)	eq(‘for’)			[@multiple]	
	… after adjusting **for age, smoking status,** and **clinical history of diabetes mellitus**.	adjusting	for			**age**, **smoking status**, and **clinical history of diabetes mellitus**.	
	**Rule**	eq(‘including’)	[@multiple]		eq(‘as’)	@synonyms	
	… including **visceral adipose tissue (vat)** and **subcutaneous adipose tissue (sat)** as covariates.	including	**visceral adipose tissue (vat)** and **subcutaneous adipose tissue (sat)**		as	covariates	
**Effect size** (15 rules)	**Rule**	@multiple	[a(preva)		a(be)	@perce]	
	Hernia **prevalence was 32.4%**	Hernia	**prevalence**		**was**	**32.4%**	
	**Rule**	@multiple	@or			@ci	
	… more likely to have **elevated blood pressure (or = 9.05, 95% ci: 1.44, 56.83)**	elevated blood pressure	**(or = 9.05,**			**95% ci: 1.44, 56.83)**	

The rule components in square brackets are the extracted spans that denote the key characteristic; the rest of the rule (if any) specifies the context. The rules use explicit matching of spans (e.g. *eq(‘onset’)*), regular expressions (*re*) for matching specific verbs or prepositions (e.g. *re(‘(of|on|in)’)*), various vocabularies that contain single (e.g. *a(types)* – matching words that indicate the conduction of a study (e.g. *study*, *analysis*, *review*)) and multiword terms (e.g. *@st*, a vocabulary of epidemiological study designs (e.g. *case control*)). *totals* contains words that suggest the participant population; *stats* is a dictionary that contains numbers and words that express numeric values (e.g., *one hundred*); *clusters* includes the variations that a population sample can be described (e.g., *men*, *patients*, *individuals*)*; multiple* contains single or multi-word biomedical concepts (e.g., *depression*, *type 2 diabetes*)*; relations* is a dictionary with single words that describe an association between concepts (e.g., *relationship, link, association*)*; factors* contains single or multi-word terms that describe risk factors (e.g., *risk factors*, *predictors*)*; or* is a dictionary that contains noun phrases in which the effect size “odds ratio” can be expressed, including the ways in which its numeric value is presented (e.g., *odds ratio = 1.34, or = 2.56*)*; ci* follows a similar pattern for confidence interval with its assigned numeric value e.g., (*95% ci = 0.91, 95% ci: 4.36, 5.48*).

## Results

### Evaluation

We evaluated the system’s performance at the document level by considering whether selected spans were correctly marked in text. We calculated precision, recall and F-score for each of the characteristic of interest using the standard definitions [31]. In order to create an evaluation dataset, 60 abstracts were randomly selected from the PubMed results obtained by query *obesity/epidemiology[mesh]* and manually double-annotated for all the six epidemiological characteristics by the first author and an external curator with epidemiological expertise. The inter-annotator agreement of 80% was calculated on the evaluation dataset by the absolute agreement rate [32], suggesting relatively reliable annotations.

Table 2 shows the results on the evaluation set, with to the results obtained on the training and development sets for comparison (Tables 3 and 4). The precision and recall values ranged from 79% to 100% and 80% to 100%, with F-measures between 82% and 96%. The best precision was observed for study design (100%). However, despite having a relatively large number of study design mentions in the training set (38 out of 60), the development and evaluation sets had notably fewer mentions and therefore the precision value should be taken with caution. Similarly, the system retrieved covariate characteristic with 100% recall, but again the number of annotated covariate concepts was low. The lowest precision was observed for outcomes (79%), while exposures had the lowest recall (80%). With the exception of study design that saw a little increase (7.7%), recall decreased for the rest of the characteristics when compared to the values on the development set. On the other hand, effect size had a notable increase in precision, from 75% (development) to 97% (evaluation). Overall, the micro F-score, precision and recall for all the six epidemiological characteristics were 87%, 88% and 86% respectively, suggesting reliable performance in the identification of epidemiological information from the literature.

**Table 2:** Results, including true positives (TP), false positives (FP), false negative (FN), precision (P), recall (R) and F-score on the evaluation set

.	**Evaluation set (60 abstracts)**					
	**TP**	**FP**	**FN**	**P**	**R**	**F**
**Study design**	12	0	1	100.0	92.3	95.9
**Population**	35	1	4	97.2	89.7	93.3
**Exposure**	45	8	11	84.9	80.3	82.5
**Outcome**	73	19	13	79.3	84.8	82.4
**Covariate**	17	2	0	89.4	100.0	94.4
**Effect size**	65	2	10	97.0	86.6	91.5
**All classes (micro)**	247	32	39	88.5	86.3	87.4
**All classes (macro)**				91.3	88.9	90.0

Micro averages are calculated across all different document level mentions; macro averages are calculated across different characteristics.

**Table 3:** Results, including true positives (TP), false positives (FP), false negative (FN), precision (P), recall (R) and F-score on the training set

.	**Training set (60 abstracts)**					
	**TP**	**FP**	**FN**	**P**	**R**	**F-score**
**Study design**	37	5	1	88.0	97.3	92.5
**Population**	94	10	5	90.3	94.9	92.6
**Exposure**	104	21	14	83.2	88.1	85.5
**Outcome**	125	26	8	82.7	93.9	88.0
**Covariate**	13	4	0	76.4	100.0	86.6
**Effect size**	41	5	9	89.1	82.0	85.4
**All classes (micro)**	414	71	37	85.3	91.7	88.4
**All classes (macro)**				84.9	92.7	88.4

Micro averages are calculated across all different document level mentions; macro averages are calculated across different characteristics.

**Table 4:** Results, including true positives (TP), false positives (FP), false negative (FN), precision (P), recall (R) and F-score on the development set

.	**Development set (30 abstracts)**					
	**TP**	**FP**	**FN**	**P**	**R**	**F**
**Study design**	11	1	2	91.6	84.6	88.0
**Population**	36	4	4	90.0	90.0	90.0
**Exposure**	59	4	0	93.6	100.0	96.7
**Outcome**	65	13	1	83.3	98.4	90.2
**Covariate**	13	3	0	81.2	100.0	89.6
**Effect size**	50	17	5	74.6	90.9	81.9
**All classes (micro)**	234	42	12	84.7	95.1	89.6
**All classes (macro)**				85.7	93.8	89.5

Micro averages are calculated across all different document level mentions; macro averages are calculated across different characteristics.

### Application to the obesity corpus

We applied the system on a large scale corpus consisting of 23,690 epidemiological PubMed abstracts returned by the *obesity/epidemiology[mesh]* query (restricted to English). We note that a number of returned MEDLINE citations did not contain any abstract, resulting in 19,188 processed citations. In total, we extracted 6,060 mentions of study designs; 13,537 populations; 23,518 exposures; 40,333 outcomes; 5,500 covariates and 9,701 mentions of effect sizes.

Table 5 shows most frequent study types in obesity epidemiological research. The most common epidemiological study designs are cohort cross-sectional (n = 1,940; 32%) and cohort studies (n = 1876; 31% of all recognized studies), whereas there were only 109 (1.7%) randomized clinical trials. Tables 6, 7, 8, 9, 10 and 11 present the most frequent exposures, outcomes and covariates along with their UMLS semantic types.

**Table 5:** The most frequent study designs extracted from the obesity epidemiological literature

**Study design**	**Frequency**	**%**
*Cross-sectional*	1,940	32.0
*Cohort*	1,876	30.9
*Review*	678	11.1
*Population/epidemiological*	521	8.5
*Case control*	341	5.6
*Observational*	191	3.1
*Non randomized controlled*	109	1.7
*Non randomized*	109	1.7
*Qualitative descriptive*	95	1.5
*Qualitative*	49	0.8

Frequency is the number of documents, and the last column presents the share within the entire set.

**Table 6:** The most frequent exposures extracted from the obesity epidemiological literature

**Exposures**	**Frequency**	**%**
*Obesity*	2,450	10.4
*Body mass index*	1,351	5.7
*Overweight*	531	2.2
*Age*	394	1.6
*Waist circumference*	291	1.2
*Physical activity*	289	1.2
*Hypertension*	256	1.0
*Metabolic syndrome*	240	1.0
*Body weight*	218	0.9
*Type 2 diabetes*	206	0.8
*Gender*	193	0.8
*Smoking*	186	0.7
*Abdominal obesity*	135	0.5
*Insulin resistance*	128	0.5
*Mortality*	117	0.4
*Adiposity*	116	0.4
*Weight gain*	108	0.4
*Diet*	98	0.4
*Childhood obesity*	92	0.3
*Weight loss*	89	0.3
*Waist to hip ratio*	82	0.3
*Education*	79	0.3
*Childhood*	79	0.3
*Socioeconomic status*	75	0.3
*Ethnicity*	75	0.3
*Depression*	70	0.2
*Central obesity*	69	0.2
*Pregnancy*	67	0.2
*Race*	66	0.2
*Blood pressure*	66	0.2
*Overweight/obesity*	59	0.2
*CVD risk factors*	59	0.2
*Height*	55	0.2
*Morbidity*	54	0.2
*Leptin*	52	0.2
*Birth weight*	49	0.1
*Asthma*	49	0.1
*Bariatric surgery*	48	0.1
*Physical inactivity*	47	0.1
*Family history*	45	0.1

Frequency is the number of documents, and the last column presents the share within the entire set.

**Table 7:** Distribution of UMLS semantic groups assigned to exposures

**Semantic group**	**Frequency**	**%**
Disorders	8,700	36.9
Concepts/ideas	4,635	19.7
Physiology	3,969	16.8
Procedures	1,611	6.8
Activities/behaviors	1,285	5.4
Living beings	1,030	4.3
Chemicals/drugs	857	3.6
Objects	368	1.5
Genes/molecular	344	1.4
Anatomy	252	1.0
Phenomena	180	0.7
Geographic areas	145	0.6
Occupations	73	0.3
Devices	30	0.01
Organizations	21	0.0
Other	16	0.0

**Table 8:** The most frequent outcomes extracted from the obesity epidemiological literature

**Outcomes**	**Frequency**	**%**
*Obesity*	5,220	12.9
*Overweight*	2,058	5.1
*Type 2 diabetes*	1,379	3.4
*Body mass index*	1,084	2.6
*Hypertension*	728	1.8
*Cardiovascular disease*	712	1.7
*Metabolic syndrome*	659	1.6
*Mortality*	460	1.1
*Insulin resistance*	297	0.7
*Childhood obesity*	289	0.7
*Coronary heart disease*	260	0.6
*Death*	250	0.6
*Health*	225	0.5
*Waist circumference*	211	0.5
*Abdominal obesity*	209	0.5
*Smoking*	194	0.4
*Physical activity*	193	0.4
*Weight gain*	181	0.4
*Morbidity*	180	0.4
*Cvd risk factors*	175	0.4
*Weight*	162	0.4
*Adiposity*	161	0.3
*Overweight/obesity*	155	0.3
*Asthma*	127	0.3
*Blood pressure*	122	0.3
*Dyslipidemia*	116	0.2
*Body weight*	110	0.2
*Stroke*	101	0.2
*Central obesity*	98	0.2
*Depression*	95	0.2
*Weight loss*	94	0.2
*Underweight*	91	0.2
*Chronic diseases*	91	0.2
*Hypercholesterolemia*	88	0.2
*Cancer*	86	0.2
*Survival*	85	0.2
*Cardiovascular risk*	85	0.2
*Atherosclerosis*	81	0.2
*Coronary artery disease*	78	0.1
*Inflammation*	68	0.1

Frequency is the number of documents, and the last column presents the share within the entire set.

**Table 9:** Distribution of UMLS semantic groups assigned to outcomes

**Semantic group**	**Frequency**	**%**
Disorders	21,809	54.0
Concepts/ideas	7,277	18.0
Physiology	3,810	9.4
Procedures	1,697	4.2
Living beings	1,616	4.0
Activities/behaviors	1,413	3.5
Chemicals/drugs	990	2.4
Anatomy	577	1.4
Objects	314	0.7
Genes/molecular	265	0.6
Phenomena	250	0.6
Geographic areas	137	0.3
Occupations	102	0.2
Organizations	36	0.0
Devices	28	0.0
Other	16	0.0

**Table 10:** The most frequent covariates extracted from the obesity epidemiological literature

**Covariates**	**Frequency**	**%**
*Age*	1,066	19.3
*Gender*	631	11.4
*Body mass index*	346	6.2
*Smoking*	260	4.7
*Education*	160	2.9
*Race*	117	2.1
*Physical activity*	108	1.9
*Alcohol consumption*	83	1.5
*Ethnicity*	70	1.2
*Type 2 diabetes*	67	1.2
*Race/ethnicity*	60	1.0
*Obesity*	58	1.0
*Waist circumference*	53	0.9
*Income*	43	0.7
*Hypertension*	42	0.7
*Socioeconomic status*	39	0.7
*Height*	36	0.6
*Marital status*	33	0.6
*Demographics*	32	0.5
*Parity*	27	0.5
*Smoking status*	25	0.5
*Energy intake*	25	0.5
*Lifestyle*	22	0.4
*Educational level*	20	0.3
*Birth weight*	20	0.3
*Weight*	17	0.3
*Maternal age*	17	0.3
*Family history*	17	0.3
*Exercise*	16	0.2
*Depression*	15	0.2
*Total energy intake*	14	0.2
*Region*	13	0.2
*Insulin resistance*	13	0.2
*Occupation*	12	0.2
*Family income*	12	0.2
*Blood pressure*	12	0.2
*Adiposity*	11	0.2
*Social class*	10	0.1
*Gestational age*	10	0.1
*Area*	10	0.1

Frequency is the number of documents, and the last column presents the share within the entire set.

**Table 11:** Distribution of UMLS semantic groups assigned to covariates

**Semantic group**	**Frequency**	**%**
Physiology	2,381	43.2
Concepts/ideas	1,044	18.9
Disorders	783	14.2
Activities/behaviors	591	10.7
Living beings	232	4.2
Procedures	184	3.3
Chemicals/drugs	112	2.0
Geographic areas	41	0.7
Occupations	34	0.6
Objects	29	0.5
Phenomena	26	0.4
Genes/molecular	17	0.3
Anatomy	17	0.3
Other	4	0.0
Organizations	4	0.0
Devices	1	0.0

## Discussion

Compared to other approaches that focused specifically on randomized clinical trials, our approach addresses a significantly more diverse literature space. We aimed at extracting key epidemiological characteristics, which are typically more complex than those presented in clinical trials. This is not surprising because clinical trials are subject to strict regulations and are reported in highly standardised ways. Although this makes it difficult to compare our results with those of others directly, we still note that our precision (79-100%) is comparable to other studies (67-93%). The overall F-score of 87% suggests that a rule-based approach can generate reliable results in epidemiological text mining despite the restrained nature of the targeted concepts. Here we discuss several challenges and issues related to epidemiological text mining, and indicate the areas for future work.

### Complex and implicit expressions

Despite having relatively reliable annotations (recall the inter-annotator agreement of 80%), epidemiological abstracts feature a number of complex, varying detail and implicit expressions that are challenging for text mining. For example, there are various ways in which population can be described: from reporting age, sex and geographical region to mentioning the disease the individuals are currently affected with or that are excluded from the study (e.g. “The study comprised of *52 subjects with histologically confirmed advanced colorectal polyps and 53 healthy controls*” [PMID – 21235114]). Even more complex are the ways in which exposures are expressed, given that these are not often explicitly stated in text as exposures but rather part of the context of the study. Similarly, identification of covariate concepts is challenging as only a small number of covariates are explicitly stated in text.

Finally, out dictionary coverage and focus were quite limited by design: we focused on biomedical concepts, but other types of concepts may be studied as determinants and outcomes, or being mentioned as covariates (e.g., “*high school environmental activity*”). While these have been addressed by application of ATR, more generic vocabularies may need to be used (see below for some examples).

### Error analysis on the evaluation dataset

Our approach is based on intensive lexical and terminological pre-processing and rules to identify the key epidemiological characteristics. The number of rules designed for obesity can be considered relatively high (412), given that they were engineered from relatively small training (and development) datasets. On one hand, the number of rules for study design (16), covariate (28) and effect size (15) were rather small in comparison to others e.g., population (119), indicating the existence of generic expression patterns that can identify concept types from more generic epidemiological characteristics (such as study design or effect size). However, disease-related concepts often include a variety of determinants along with a number of outcomes of various nature (e.g. anatomical, biological, disease-related, etc.). Therefore, on the other hand, the task of recognizing these epidemiological elements (e.g., outcomes, exposures) through a rule based approach is not an easy task and requires a number of rules to accommodate different types of expression. We briefly discuss the cases of errors for each of the characteristic below.

#### Study design

Due to the limited number of study design mentions (only 13) in the evaluation set, the high values of precision, recall and F-score should be taken with caution. There were no false positives in the evaluation data set. However, it is possible that in a larger dataset, false positives could appear if certain citations report more than one mention of different study types. In addition, study designs without specific information can be ambiguous and thus were ignored (e.g. “*Metabolic and bariatric surgery for obesity: a* ***review*** [False Negative]”).

#### Population

An analysis of false positives reveals that rules relying on the identification of prepositional phrases associated with populations (e.g. *among* and *in*) need more specific presence of patient-related concepts. False negatives included “*3,715 deliveries*” or “*895 veterans who had bariatric surgery*”, which are referring to births and a specific demographic respectively, but our lexical resources did not contain those. Nevertheless, the F-score for the population type was the second best (93%), showing that a rule-based approach can be used to identify the participants in epidemiological studies. An interesting issue arose in the identification of population associated to meta-analyses. For example, the mention “*included 3 studies involving 127 children*” was identified by patterns but it is clear that a specific approach would be needed for meta-analysis studies.

#### Exposures and outcomes

While outcomes are often explicitly mentioned in text as such, exposure concepts are not, which makes the identification of exposures a particularly challenging task. Still, the use of dictionaries containing biomedical concepts for identification of potential mentions proved useful for capturing exposure concepts. However, dictionary-based look-up also contributed to incorrect exposure candidates that were extracted from non-relevant contexts. On the other hand, two frequent causes of errors could be linked to missing concepts from our dictionaries (e.g. “*late bedtimes*” or “*costs*”) and relatively complex exposure expressions (e.g. “*level of PA during leisure*”).

An important source of errors was the confusion between exposures and outcomes, given they both refer to similar (semantic) types whose instances can – in different studies – be either exposure or outcome, and thus their role can be easily misinterpreted as an outcome rather than a studied determinant (and vice versa). We noted that rules such as “*association between < exposure > and < outcome>”* or *“<exposure > associated with < outcome>”* generated encouraging results i.e., a number of TPs. This was not surprising: when a clinical professional is studying the relationship between two concepts, he explores the link between an exposure and an outcome, which the above patterns capture. Still, sometimes these patterns would match links irrelevant to exposure/outcome relationships (e.g. “*relationship between race and gender*”). Cases like these result in the generation of both false positives and false negatives. Overall, a sentence-focused rule based method may struggle to understand a concept’s role in a given case, and a wider context might need to be considered.

#### Covariates

Covariates had only a limited number of identified spans, hence any conclusion regarding the system’s performance is at most indicative. Still, the results could provide an initial indication that (at least explicit) covariate mentions could be detected with good accuracy, despite some false positives (e.g. a generic mention “*potential confounders*” was identified as a covariate in “*… after adjustment for potential confounders*”).

#### Effect size

The rules designed to recognize effect size spans were based on the combination of numerical and specific lexical expressions (e.g. “*relative risk*”, “*confidence interval*”). A relatively high recall (87%) revealed that this approach returned promising results, with only a small number of mentions being ignored by the system, but with high precision. False negatives included expressions that included multiple values (e.g., “*… increased risks of overweight/obesity at the age of 4 years (odds ratio (95% confidence interval): 15.01 (9.63, 23.38))*”, “*… bmi statistically significantly increased by 2.8% (95% confidence interval: 1.5% to 4.1%; p < 0.001) …*”).

### Application to the obesity corpus

Although we had relatively good recall in both the development and evaluation datasets, the experiments with the entire obesity dataset have shown that the system extracted epidemiological information only from a limited number of documents. We have therefore explored the reasons for that.

#### Study design

We identified study type from only around 40% of processed articles (each tagged as *obesity/epidemiology*). To explore whether those missed study design mentions are due to our incomplete dictionaries and rules, we inspected 20 randomly selected articles from those that contained no identified study type, and we identified the following possible reasons:

• **No mention of study design**: while the article presents an epidemiological context, no specific epidemiological study had been conducted (and thus there was no need to specify study design) – this was the case in almost 2/3 of the abstracts with no study design;

• **Summarised epidemiological studies**: articles summarizing epidemiological information but without reporting a specific conducted study and its findings (15% of the abstracts);

• **Other study designs**: studies including comparative studies, surveys, pilot studies, follow-up studies, reports, reviews that were not targeted for identification (20% of the abstracts).

We note that we can see a similar pattern in the evaluation dataset (which was randomly selected from the obesity corpus). Importantly, for the majority of abstracts in the evaluation dataset, if the system was able to detect the study type, all other epidemiological characteristics have been extracted with relative success, providing a complete profile of an epidemiological study (data not shown).

#### Covariates

Only 5,500 confounding factors were recognised. To explore the reason for so many articles not having covariates extracted, a random sample of 20 abstracts in which no covariate concept was identified was investigated. None of the studied abstracts contained any covariate mentions. Most abstracts used only generic expressions (e.g., *“after adjustment for confounding factors”*, *“after controlling for covariates”*) without specifying the respective concepts. We note that we only processed abstracts and it seems likely that covariates may be defined in full-text articles.

#### Effect size

Similar observations to the ones made for the covariate characteristic were noted for the effect size mentions (only 9,701 mentions were extracted). We explored a sample of 20 abstracts in which no effect size was recognised. As many as 60% of the abstracts did not report any observed effect size between the studied exposures and outcomes due to the nature of the conducted study (e.g. pilot study, systematic review, article). We failed, however, to get effect size mentions in 40% of cases, mainly because of mentions that contained coordinated expressions (e.g. “*The prevalence of hypertension was considerably higher among men than among women (60.3% and 44.6%, respectively*”; PMID 18791341) or statistical significance data, which are not covered by our rules.

#### Outcomes

As opposed to other characteristics, the number of recognised outcome concepts was more than double the number of abstracts. This is not a surprise, as most of the epidemiological studies include more than one outcome of interest. In addition, with the current system, we have not attempted to unify synonymous terms (unless they are simple orthographic variants).

## Conclusions

We presented a generic rule based approach for the extraction of the six key characteristics (study design, population, exposure(s), outcome(s), covariate(s) and effect size) from epidemiological abstracts. The evaluation process revealed promising results with the F-score ranging between 82% and 96%, suggesting that automatic extraction of epidemiological elements from abstracts could be useful for mining key study characteristics and possible meta-analysis or systematic reviews. Also, extracted profiles can be used for identification of gaps and knowledge modelling of complex health problems. Although our experiments focused on obesity mainly for the purpose of evaluation, the suggested approach of identifying key epidemiological characteristics related to a particular clinical health problem is generic.

Our current work does not include identification of synonymous expressions or more detailed mapping of identified terms to existing knowledge repositories, which would allow direct integration of the literature with other clinical resources. This will be the topic for our future work. Another potential limitation of the current work is that we focused only on abstracts, rather than full-text articles. It would be interesting to explore if full-text would improve the identification (in particular recall) or it would introduce more noise (reducing precision).

## Availability and requirements

Project name: EpiTeM (Epidemiological Text Mining)

Project home page: http://gnode1.mib.man.ac.uk/epidemiology/

Operating system(s): Platform independent

Programming language: Python

Other requirements: MinorThird

License: FreeBSD

Any restrictions to use by non-academics: None

## Abbreviations

ATR: Automatic term recognition; FN: False negatives; FP: False positives; P: Precision; R: Recall; RCT: Randomized clinical trial; TP: True positives; UMLS: Unified Medical Language System.

## Competing interests

The authors declare that they have no competing interests.

## Authors’ contributions

The study was conceived and designed by IB and GN. GK implemented the system, provided the data and performed the experiments. All authors read and approved the final manuscript.
